# Speaking Rate, Oro-Laryngeal Timing, and Place of Articulation Effects on Burst Amplitude: Evidence From English and Tamil

**DOI:** 10.1177/00238309221133836

**Published:** 2022-12-08

**Authors:** Chandan R. Narayan

**Affiliations:** Speech and Psycholinguistics Lab, Department of Languages, Literatures & Linguistics, York University, Canada

**Keywords:** Burst amplitude, voice-onset time, speaking rate, Tamil, English

## Abstract

The relationship between speaking rate and burst amplitude was investigated in plosives with differing oro-laryngeal timing: long-lag voice-onset time (VOT) (North American English) and short-lag VOT (Indian Tamil). Burst amplitude (reflecting both intraoral pressure and flow geometry of the oral channel) was hypothesized to decrease in pre-vocalic plosive syllables with the increase in speaking rate, which imposes temporal constraints on both intraoral pressure buildup behind the oral occlusion and respiratory air flow. The results showed that decreased vowel duration (which is associated with increased speaking rate) led to decreased burst amplitude in both short- and long-lag plosives. Aggregate models of bilabial and velar plosives (found in both languages) suggested lower burst amplitudes in short-lag stops. Place-of-articulation effects in both languages were consistent with models of stop consonant acoustics, and place interactions with vowel duration were most apparent with long-lag English stops. The results are discussed in terms of speaking rate and language-internal forces, contributing to burst amplitude variation and their implications for speech perception and potential to affect lenition phenomena.

## 1 Introduction

### 1.1 Background

Speaking rate is a highly variable phenomenon affected by a host of linguistic and extra-linguistic factors, resulting in adjustments to articulatory targets (Gay, 1981) and velocities (Adams et al., 1993; Dromey & Ramig, 1998). The phonetic consequences of modulations of speaking rate broadly affect both consonant temporal characteristics (e.g., voice-onset time [VOT]; Kessinger & Blumstein, 1997) and spectral properties (e.g., vocalic trajectories; Lindblom, 1983). There has been considerable attention given to the phonetic effects of speaking rate in the context of (non-phonologized) consonant *lenition*, with much of the literature focused on intervocalic lenition processes in a variety of languages as indexed by consonant intensity (Priva & Gleason, 2020; Warner & Tucker, 2011; see Lavoie, 2001 for a review) and consonant duration. Intensity (or root-mean-square [RMS] amplitude) in this literature is a measure of periodic, sonorous energy in intervocalic consonants often without complete closure and lacking the release characteristics of initial plosives. The general pattern is for increased speaking rate (either implemented directly or as a consequence of style or register shifts) to result in increased intensity in intervocalic consonants and reduced consonant duration (Priva & Gleason, 2020; Soler & Romero, 1999; Warner & Tucker, 2011; Winter & Grawunder, 2012). Lenition of this sort affects perception (Tucker, 2011) such that words with phonetically reduced variants are difficult to process. While a clear picture is emerging of how speaking rate modulates the acoustic characteristics of postvocalic and intervocalic consonants, very little is known about the relationship between speaking rate and the amplitude characteristics of initial prevocalic consonants, and in particular their release burst.

### 1.2 Obstruent bursts and extra-linguistic variation

The release of an oral stop consonant results in transient energy across the frequency spectrum, which is generally followed by broadband noise, collectively taken as the “burst.” The transient portion of the burst, reflecting the release of pressurized intraoral air posterior the oral occlusion, together with the noise or aspiration following the transient, reveals the resonant properties of the tube anterior the oral occlusion being excited by air escaping the lungs prior to phonation. For example, velars generally have a mid-frequency prominence as a result of a relatively long oral cavity. Indeed much of the literature does not examine burst variation directly, but rather intraoral pressure (*P*_o_), which serves as a proxy measure suggesting possible effects on burst characteristics.

Although a buildup of intraoral pressure *P*_o_ is necessary for the plosive burst characterizing oral stop consonants, it is subject to naturally occurring variation caused by extra-linguistic factors like stress and emotional state (Laukkanen et al., 1996) or affective voice quality such as whispering (Murry & Brown, 1976). While the extra-linguistic feature of speaking rate would seem, at first blush, to affect *P*_o_ (and by proxy, release bursts), models of *P*_o_ for voiceless stops suggest that it is not straightforwardly affected by the temporal constraints imposed by increased rate. Müller & Brown (1980) (following Rothenberg, 1966) show *P*_o_ in voiceless stops has a rise time on the order of 50 ms (beginning before complete oral occlusion), which is potentially sufficient time to achieve *P*_o_ maxima in speeded speaking rate conditions where closure duration at fasts rates are generally greater than 50 ms (Allen & Miller, 1999; Pickett et al., 1999). Nonetheless, some studies suggest that *P*_o_ is indeed correlated with speaking rate. In a study of Swedish aspirated “pa” spoken by a single speaker at different rates (1–3 syllables/s), Hertegård et al. (1995) found a high correlation between subglottal pressure and *P*_o_, which decreased by approximately 4–5 cm H_2_O at fast speaking rates. C. J. Miller and Daniloff (1977) examined *P*_o_ in three speakers’ productions of English CVCs in monologues (thought to be comparable to conversational speaking rate) and citation contexts. For all three speakers, *P*_o_ for voiceless stops was at least 1 cm H_2_O lower in monologue than citation contexts. Speaking rate also affects physiological properties of respiration. Lung volumes in speeded speaking tasks are lower than in slow speech (Dromey & Ramig, 1998), which in turn results in lower subglottal pressure (Sundberg, 2018) roughly corresponding to *P*_o_ during complete closure in plosive production.

Given the potential effect of speaking rate variation on *P*_o_, comparable effects on burst amplitude are expected; however, there is a gap in the literature directly addressing this question. There is some indirect evidence on the relationship between speaking rate and burst amplitude, which shows mixed effects. In their study of *P*_o_ and speaking style, C. J. Miller and Daniloff (1977) also reported on peak absolute sound pressure level at consonant release, which showed mixed results between the monologue- and citation-style consonant bursts. Burst amplitude was an absolute measure of the maximum sound pressure level in the RMS trace of release. One of the three speakers showed a correlated effect of speaking style on burst amplitude, which was lower in monologue speech (presumably articulated at a faster rate) than citation in all three places of articulation. The two other speakers exhibited varying effects of speaking style. The authors note that in their data, burst amplitude was correlated with both *P*_o_ and closure duration in two speakers, suggesting that it is not straightforwardly a reflection of *P*_o_ and that aerodynamic variables along with additional physiological parameters must be incorporated into models of normal speech. Indeed speakers’ passive expansion of the oral cavity (e.g., depressing tongue body, expanding cheeks) and tissue compliance have been shown to slow the buildup of *P*_o_ during stop closure, and are highly variable both within and across speakers, thereby contributing to the complex effects on burst amplitude (Bell-Berti, 1975; Westbury, 1983).

### 1.3 Obstruent bursts and linguistic variation

Burst amplitude varies according to phonological features of voicing and place of articulation. Burst amplitudes in phonologically voiceless stops are generally higher than for voiced stops (Chodroff & Wilson, 2014; Stevens et al., 1999), reflecting greater intraoral pressure (*P*_o_) and airflow at consonant release (Haag, 1977; Subtelny et al., 1966). Voicing in initial obstruents, which has as its primary articulatory implementation the synchronization of oral and laryngeal gestures, necessarily affects the pressurized air in the oral cavity (reflected in burst amplitude), but also results in corresponding phonetic characteristics like vocal fold oscillation prior to closure release, depressed fundamental frequency in the following vowel, and so on (Lindqvist, 1972; Kingston & Diehl, 1994), all of which potentially affect *P*_o_, and consequently, burst amplitude. While burst amplitude is affected by the laryngeal feature of voicing, there is some limited evidence that it may not be affected by the fortis-lenis contrast (DiCanio, 2012).

Place of articulation affects the shape and amplitude of the burst. The spectral envelope reflects the length of the cavity anterior the oral closure (e.g., no cavity for bilabials, short cavity for alveolars, and long cavity for velars) and resonances (Stevens et al., 1999). Place of articulation affects burst amplitude as well, generally reflecting *P*_o_ at varying cavity volumes and rates of airflow after closure release, with bilabials having the lowest amplitudes (Stevens, 1998; Stevens et al., 1999). Importantly, both spectral envelope and amplitude characteristics of the release burst in certain frequency bands have been shown to be perceptually relevant for both place of articulation and voicing, respectively (Blumstein & Stevens, 1979; Chodroff & Wilson, 2014; Krull, 1990; Ohde & Stevens, 1983; Repp & Lin, 1989; Winitz et al.,1972).

### 1.4 Research goals

The goals of the present research are to (1) test the hypothesis that burst amplitude in CV syllables decreases with the increase in speaking rate, (2) examine whether such speaking rate effects are more or less evident at various places of articulation, and (3) explore the interaction between speaking rate and oro-laryngeal synchronization (which is phonetically implemented in terms of short- and long-lag VOT) on burst amplitude. It is hypothesized that burst amplitude is related to VOT, with long-lag VOT being associated with high *P*_o_ and burst amplitude. The faster initiation of vocal fold oscillation in short-lag VOTs suggests that *P*_o_ is sufficiently low to promote enough of a trans-glottal pressure differential for the rapid onset of phonation. This would be manifested in lower burst amplitude. The study focuses on consonants broadly classified as “voiceless,” that is, consonants where the primary acoustic and perceptual characteristic is VOT. Previous research on speaking rate, *P*_o_, and burst amplitude was conducted in languages (English and Swedish), where pre-vocalic word-initial voiceless plosives are necessarily aspirated. The present study examines the relationship between relative burst amplitude and speaking rate in speakers of North American English (Indo-European) and Indian Tamil (Dravidian), a language which lacks phonemic voicing and exhibits short-lag VOT word-initial plosives (Keane, 2004).

In comparing short-lag (Tamil) and long-lag (English) plosives, the present study addresses the contribution of speaking rate and oro-laryngeal timing dynamics to observed burst amplitude effects. Short-lag plosives from Tamil are examined instead of English short-lag stops (the voiced series /b d g/ [b° d° g°]) to avoid aerodynamic and acoustic characteristics of phonologically voiced stops (such as closure voicing/voicing lead variability, passive cavity expansion during closure voicing, vocal fold adduction variability, *F*_0_ perturbation; Docherty et al., 2011; Flege, 1982; Kingston & Diehl, 1994; Lindqvist, 1972; Lisker, 1986) and associated *P*_o_ variability which might potentially obscure the effects of VOT on burst amplitude. These phonetic characteristics associated with English have not been reported for short-lag Tamil plosives, where there is no phonological contrast. Given that the hypothesized mechanism affecting burst amplitude variation in the current study is *P*_o_ constrained by time in speeded conditions, the short-lag series from Tamil, rather than the phonologically voiced English series, was considered a more appropriate comparison with the long-lag English series. Language differences are, therefore, explained in terms of the effects of VOT type on burst amplitude.

This more general effect of the temporal constraints imposed by speaking rate may have been obscured in previous research by an absolute burst amplitude measure (C. J. Miller & Danilo, 1977). In this study, we examine the effect of speaking rate on relative measures of burst amplitude, where peak amplitudes in various bands of burst noise are analyzed in relation to the amplitude of the following vowel. Such a normalization accounts for the overall amplitude of the utterance and may be a better index of the perceptual adjustments made by listeners when assessing place of articulation (Ohde & Stevens, 1983).

Finally, the results of the study are discussed in the context of broader sources of variation, both language-internal (oro-laryngeal synchrony) and extra-linguistic (speaking rate), and directions for future study examining potential perceptual effects of burst amplitude variation are offered.

## 2 Method

### 2.1 Speakers

The speech of 12 North American (Canadian and American) English speakers (six female and six male), aged 36–45 years, and 10 Indian Tamil speakers (five female and five male), aged 24–44 years, was analyzed. All participants were native speakers of their respective languages, with no reported speech or hearing disorders. Participants were recruited via email solicitation and through various social media platforms.

### 2.2 Procedure

Due to restrictions to laboratory access during the global pandemic, participants were asked to record themselves in a quiet room at their respective residences (in Canada, America, and India). Participants were instructed to use a wired microphone (either ear-bud or closed-ear gaming style) along with recording software (such as Audacity or Praat) installed on their home computer. English-speaking participants were instructed to make three recordings, one each for the monosyllables “pa,” “ta,” and “ka.” For each recording, participants were asked to repeat the syllable at four self-paced speaking rates (slow, normal, fast, very fast) for approximately 5 s per rate. Instructions for Tamil-speaking participants were identical but with recordings for four places of articulation, bilabial ([pa]), dental ([t⊓a]), velar ([ka]), and retroflex ([ʈa]). Instructions included an example audio file of the syllable “pa” (American English as recorded by the author) modeling the rate manipulation and providing a general structure of the recording. Participants made recordings with sampling rates at or above 22 kHz and saved as .wav or .aiff files. Given the nature of the instructions (5 s per rate) participants produced more fast-rate tokens than slower rate tokens. There was some concern that amplitude measurements would be affected by the variety of microphones and recording interfaces employed by the participants. No post hoc scaling was performed on raw amplitude measurements (burst) as difference measures (between the burst and adjacent vowel) served as dependent variables (see Section 2.3).

### 2.3 Measurements

All measurements were made by hand using Praat speech processing software by trained phoneticians. Two temporal measurements were made: duration of consonant release/burst (VOT), and vowel duration. The onset of consonant release was identified at the first appearance of broadband transient noise, which was often followed by a burst of frication noise. Both transient and frication were considered the “burst.” Burst offset was identified at the zero-crossing before the first glottal cycle of the following vowel. The vowel onset (coinciding with the burst offset) was indexed by the first low-amplitude periodic oscillation. The end of the vowel (in the CV syllables) was marked by three co-occurring events: (1) a dramatic change in amplitude in the waveform, (2) a change in the energy in the formants accompanied by a change in complexity in the waveform indicating a loss of energy in F2 and F3, and (3) the onset of aperiodicity.

**Figure 1.**
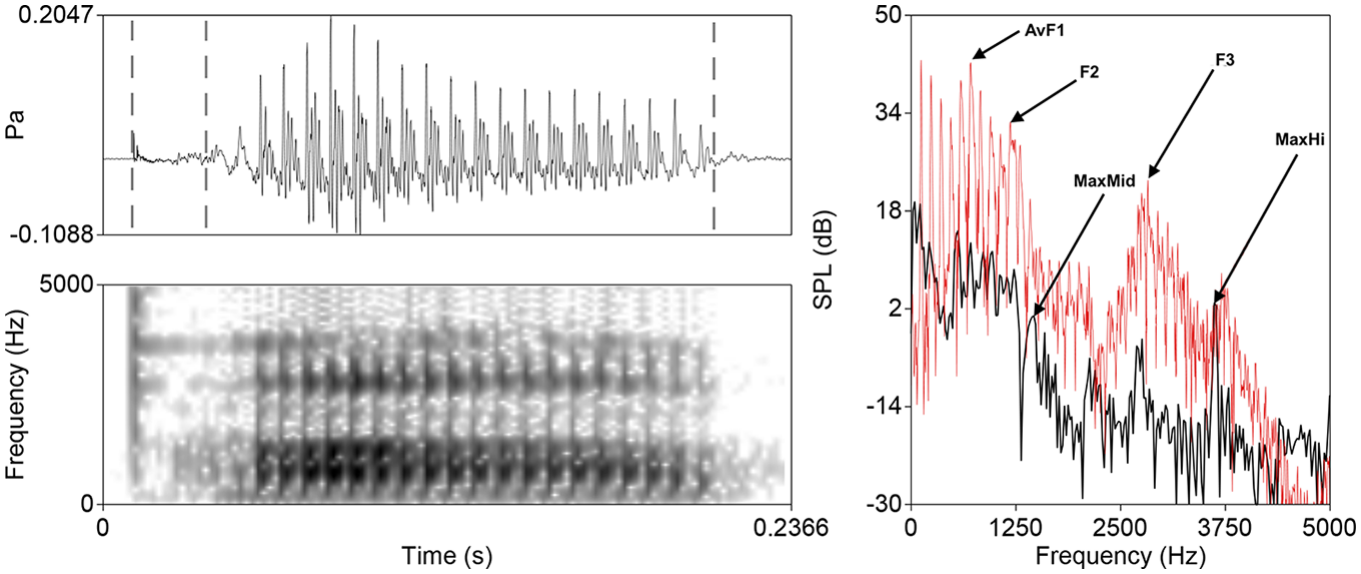
Waveform and wide-band spectrogram representations (left) of a typical “pa” token spoken by a male North American English speaker and averaged spectra for the burst and vowel (right). Three dashed lines on the waveform represent burst onset, burst offset/vowel onset, and vowel offset. Critical spectral measurements described in Section 2.3 are indicated on the burst spectrum (black) and vowel spectrum (red).

Following Stevens et al. (1999), the spectrum of the burst was measured using an averaging technique with a 5 ms Hamming window. The onset of periodic glottal oscillation of the vowel was avoided in the averaging window. In cases where consonant release was immediately (<5 ms) followed by glottal oscillation (generally in Tamil bilabial recordings), the unique measurement window was centered at the zero-crossing immediately preceding the onset of voicing. As one of our questions was motivated by potential place-specific effects of speaking rate–induced burst amplitude variation, three amplitude measurements were taken reflecting important frequency bands for place perception following (Ohde & Stevens, 1983): (1) **AvF1**, or the average amplitude of F1 in the vowel; (2) **MaxHi**, or the maximum spectrum amplitude in the burst above 3 kHz (for males) and 3.5 kHz (for females); and (3) **MaxMid**, or the maximum spectrum amplitude in the burst in the F2/F3 range. Following Stevens et al. (1999), which identified two regions in the burst spectrum important for place perception, two relational measures were computed for each token, high-frequency **HiDiff** (AvF1-MaxHi), and mid-frequency **MidDiff** (AvF1-MaxMid) burst amplitudes. Relational measures of burst amplitude have been used in other studies examining strength (DiCanio, 2012) and provide a token-specific normalization that mitigates the potential effect of varying microphone fidelity. Figure 1 shows the waveform and spectrogram representations of a typical token along with average spectra of the burst and vowel portions.

## 3 Results

### 3.1 Speaking rate and vowel duration

**Figure 2.**
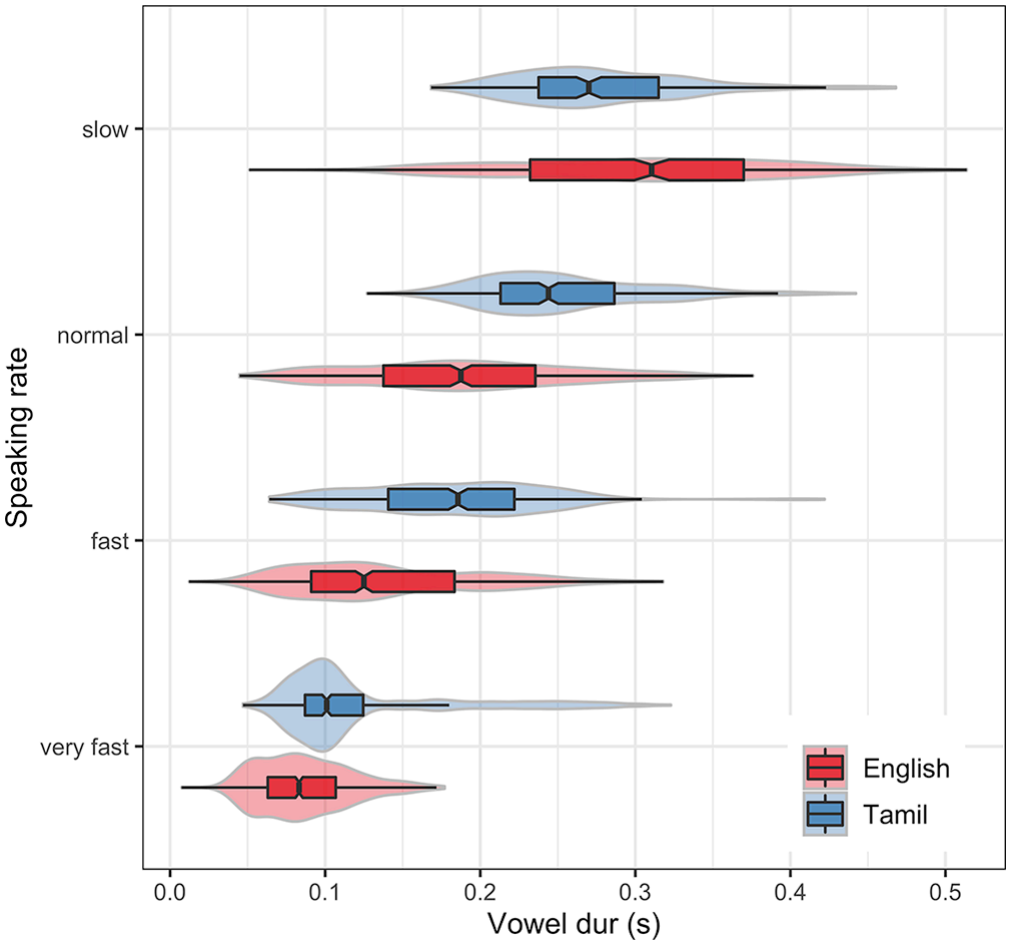
Observed vowel duration according to speaking rate and language. Violin plots show the density of vowel durations, while notched boxplots give medians and interquartile range.

Although some literature on speaking rate follows from a controlled implementation of speaking rate regulation procedures, where participants are instructed to synchronize their production with a metronome ensuring a consistent implementation of rate (e.g., J. L. Miller & Baer, 1983), much of the literature on rate effects proceed in a way similar to the present study, where participants vary their speaking speed in a subjective manner (e.g., Kessinger and Blumstein, 1997; Beckman et al., 2011). The modeling strategies in this literature rely on collapsing rate-dependent critical measurements (Gay, 1978; Kessinger & Blumstein, 1997; Soler & Romero, 1999). For example, dependent variables are analyzed as a function of a categorical independent rate variable with levels such as “slow,” “fast.” The present study is motivated by the question of acoustic consequences of durational shortening as induced by speaking rate, and as such, variability along the vowel duration *continuum*, treating it as a continuous variable. For this reason, vowel duration is taken as a proxy for subjective speaking rate, that is, it is assumed that faster speech results in shorter syllabic durations—but this may not necessarily be the case (e.g., speakers may vary the duration of inter-syllable intervals and thereby achieve slower or faster rates). To confirm this assumption, the relationship between subjective speaking rate and vowel duration was examined. The raw vowel duration data according to speaking rate and language are shown in Figure 2.

A linear mixed-effects model (nlme) was fit to the vowel duration data with rate (slow, normal, fast, very fast) crossed with language (English, Tamil) as predictors. The model was fit with random slopes and intercepts for rate by subject. Model coefficients are given in Table 1.

**Table 1.** Mixed-Effects Model of Vowel Duration as a Function of Subjective Speaking Rate and Language.

Predictor	β	*SE*	*df*	*t*
(Intercept)	0.31	0.02	3,762	17.03**
Rate_Normal_	−0.11	0.01	3,762	−8.06**
Rate_Fast_	−0.18	0.02	3,762	−8.94**
Rate_Very fast_	−0.23	0.02	3,762	−11.39**
Language_Tamil_	−0.03	0.03	19	−1.20
Rate_Normal_ × Language_Tamil_	0.09	0.02	3,762	4.13**
Rate_Fast_ × Language_Tamil_	0.09	0.03	3,762	2.81**
Rate_Very fast_ × Language_Tamil_	0.07	0.03	3,762	2.29*

*Note*. References are Rate_Slow_ and Language_English_.

** *p* < 0.01; *p < 0.05.

The model suggests that subjectively implemented speaking rate adjustments affect vowel duration in the direction predicted, increasing with the decrease in subjective rate. The interaction between rate and language reflects the longer mean vowel duration for slow rate in English speakers than Tamil speakers, which flips the more general trend in the data, where vowel durations in Tamil are longer than in English in normal, fast, and very fast rates.

To determine the effectiveness of incremental speaking rate increases in each language, separate linear models were built for English and Tamil. Table 2 shows the mixed model coefficients for each language, while Table 3 shows pairwise comparisons from estimated marginal means computed using the emmeans package.

**Table 2.** Mixed-Effects Model of Vowel Duration as a Function of Subjective Speaking Rate in (A) English and (B) Tamil.

A: English					B: Tamil			
Predictor	β	*SE*	*df*	*t*	β	*SE*	*df*	*t*
(Intercept)	0.31	0.02	2,379	14.48**	0.28	0.02	1,383	19.12**
Rate_Normal_	−0.11	0.02	2,379	−7.28**	−0.02	0.01	1,383	−1.84
Rate_Fast_	−0.18	0.02	2,379	−9.29**	−0.09	0.02	1,383	−3.83**
Rate_Veryfast_	−0.23	0.02	2,379	−12.14**	−0.16	0.03	1,383	−6.26**

*Note*. Reference level is Rate_Slow_.

** *p* < 0.001.

**Table 3.** Pairwise Comparisons of Subjective Speaking Rates from Vowel Durations in (A) English and (B) Tamil.

A: English					B: Tamil			
Contrast	Estimate	*SE*	*df*	*t* ratio	Estimate	*SE*	*df*	*t* ratio
slow–normal	0.11	0.02	2,379	7.29**	0.03	0.01	1,383	1.84
slow–fast	0.18	0.02	2,379	9.29**	0.09	0.02	1,383	3.84**
slow–very fast	0.23	0.02	2,379	12.15**	0.16	0.03	1,383	6.26**
normal–fast	0.07	0.01	2,379	6.19**	0.07	0.02	1,383	4.35**
normal–very fast	0.12	0.01	2,379	9.30**	0.13	0.02	1,383	5.82**
fast–very fast	0.05	0.01	2,379	6.01**	0.06	0.01	1,383	4.41**

** *p* < 0.001.

Pairwise comparisons of rate effects on vowel duration in English speakers showed significant differences between all rates, suggesting shortening with each successive increasing rate. The difference in vowel duration between slow and normal rates in Tamil speakers was small and not significantly different, whereas all other rates showed significant differences in the expected direction.

### 3.2 Vowel duration effects on burst amplitude

Vowel duration was used as a stand-in for speaking rate in all of the following analyses. Linear mixed-effects models were fit to examine (1) short-lag (Tamil) and long-lag (English) VOT differences in the effect of vowel duration on MidDiff and HiDiff across all places of articulation and (2) the language-specific interaction effects of vowel duration and place of articulation on MidDiff and HiDiff. All models were fit with random slopes and intercepts for vowel duration by subject.

**Figure 3.**
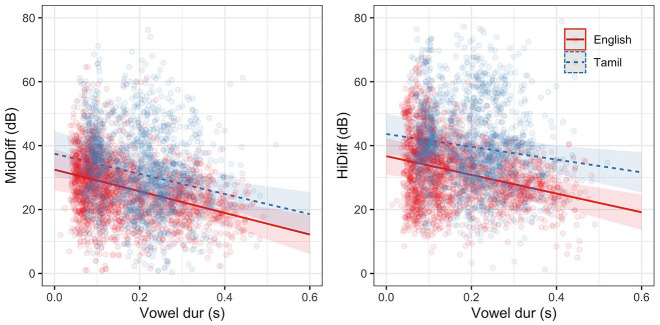
Predicted estimates (with confidence intervals) from linear mixed-effects models and observed values of MidDiff and HiDiff in long-lag (English) and short-lag (Tamil) plosives.

Model coefficients for both MidDiff and HiDiff are given in Table 4. The models show an overall negative effect of vowel duration on burst amplitude measures: with the increase in vowel duration, the difference in amplitude between the consonant and vowel decreases (∼). The models also show a small (though not significant) language difference with short-lag (Tamil) burst amplitudes being lower in the high-frequency band (*t* = 1.58, *p* = 0.12) than long-lag (English) burst amplitudes. Figure 3 shows the observed amplitude measurements as well as the model predictions for each language.

**Table 4.** Mixed-Effects Model of Relative Amplitude of Stop Bursts in (A) the F2/F3 Region (MidDiff) and (B) Above 3–3.5kHz (HiDiff), in English and Tamil.

A: MidDiff					B: HiDiff			
Predictor	β	*SE*	*df*	*t*	β	*SE*	*df*	*t*
(Intercept)	32.48	3.31	3,484	9.82**	36.71	2.93	3,484	12.55**
Vowel dur	−33.81	8.88	3,484	−3.81**	−29.27	7.15	3,484	−4.10**
Lang_Tamil_	4.96	4.96	20	0.99	6.92	4.40	20	1.58
Vowel dur × Lang_Tamil_	2.37	13.47	3,484	0.18	9.29	10.95	3,484	0.85

*Note.* Reference level is Language_English_.

** *p* < 0.001.

The results confirm the hypothesis that temporal constraints on articulation negatively affects the amplitude of release bursts in word-initial stops—with the increase in vowel duration, the difference in amplitude between the consonant burst and the following vowel decreases, or put another way, with the increase in vowel duration consonant bursts increase in amplitude. Model estimates also suggest that short-lag Tamil burst amplitude may be lower than in long-lag English plosives, especially for the high-frequency prominence at vowel durations longer than 200 ms where confidence intervals no longer overlap. The language/VOT difference may be obscured by the differing consonant inventories in English and Tamil. To explore the possible language/VOT differences on burst amplitude, the next section models the effect of vowel duration on a subset of the data where English and Tamil share consonant places of articulation—bilabial and velar plosives.

### 3.3 Place-of-articulation and vowel duration effects on burst amplitude

To tease apart possible differences between short-lag (Tamil) and long-lag (English) plosives, a subset of the full data with only bilabial and velar stops was analyzed (shared by both Tamil and English). Fully crossed models (Vowel Duration × POA × Language) of MidDiff and HiDiff were fit to the bilabial-velar data with random slopes and intercepts for vowel duration by subject. Table 5 gives model coefficients for both MidDiff and HiDiff.

**Table 5.** Linear Mixed-Effects Models of Relative Amplitude of English and Tamil Stop Bursts in (A) the F2/F3 Region (MidDiff) and (B) Above 3–3.5kHz (HiDiff), as a Function of Vowel Duration and Consonant Place of Articulation.

A: MidDiff					B: HiDiff			
Predictor	β	*SE*	*df*	*t*	β	*SE*	*df*	*t*
(Intercept)	39.67	2.64	2,064	15.03**	46.98	2.35	2,064	20.03**
Lang_Tamil_	8.12	4.01	20	2.02 +	6.89	3.61	20	1.91
POA-k	−16.60	0.67	2,064	−24.92**	−11.93	0.69	2,064	−17.37**
Vowel duration	−45.21	6.64	2,064	−6.80**	−55.99	8.30	2,064	−6.74**
Lang_Tamil_ × POA-k	−6.05	1.39	2,064	−4.37**	−4.05	1.43	2,064	−2.83*
Lang_Tamil_ × Vowel dur	15.85	10.48	2,064	1.51	43.36	12.90	2,064	3.36**
POA-k × Vowel dur	22.37	3.37	2,064	6.64**	32.63	3.48	2,064	9.39**
Lang_Tamil_ × POA-k × Vowel dur	−10.75	6.51	2,064	−1.65	−27.73	6.71	2,064	−4.13**

*Note*. References are POA-p and Lang_English_.

** *p* < 0.001; *p < 0.01; +*p* = 0.05.

The model coefficients suggest that short-lag (Tamil) burst amplitudes (for bilabials and velars) are lower (higher difference measures, βs ∼ 7–8 dB in both the mid- and high-frequency prominences) than in long-lag (English) plosives (MidDiff: *t* = 2.02, *p* = 0.05; HiDiff: *t* = 1.91, *p* = 0.07). The effect of vowel duration on burst amplitude is comparable to the models including all places of articulation in Section 3.2.

**Figure 4.**
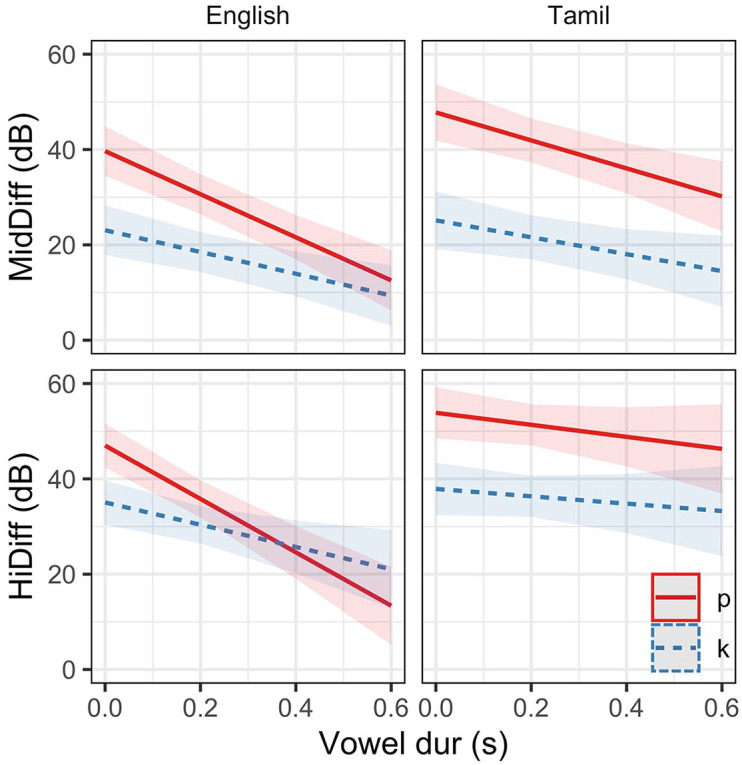
Predicted estimates (with confidence intervals) from linear mixed-effects models of MidDiff and HiDiff in bilabial and velar places in long-lag (English) and short-lag (Tamil)plosives.

Figure 4 gives the vowel duration varying model estimates of MidDiff and HiDiff for bilabials and velars in English. For the model of MidDiff burst amplitude, the relationship between bilabial and velar was different in Tamil relative to English. The interaction between POA and vowel duration suggests that vowel duration has a different effect on velars than bilabials across both languages, and the three-way interaction term, although not significant (*t* =−1.65, *p* = 0.098), suggests the effect of vowel duration on the relationship between bilabials and velars is different in the two languages (as evidenced by a slightly steeper slope for bilabials in English).

In the HiDiff model, the effect of vowel duration (across both POAs) on burst amplitude was different in short-lag (Tamil) relative to long-lag (English) plosives. The model also shows that the relationship between bilabial and velar was different in short-lag (Tamil) relative to long-lag (English). There was a significant interaction between POA and vowel duration suggesting that vowel duration has a different effect on velar burst amplitude than bilabial burst amplitude across both laryngeal specifications (English and Tamil). Finally, the three-way interaction term suggests differing effects of vowel duration on the relationship between bilabials and velars in the two languages/laryngeal specifications.

The effect of vowel duration on the various places of articulation in the two languages (with language-specific models) is explored in the next section.

### 3.4 Language-specific place-of-articulation effects on burst amplitude

Place-of-articulation effects in English and Tamil were analyzed separately to account for their differing stop-consonant inventories. Although both languages share bilabial and velar places, coronal places vary—Tamil with two, dental ([t⊓]) and retroflex ([ʈ]). Linear models of burst amplitude were fit with place of articulation and vowel duration as predictors and the same random-effects structure as the aggregate models above.

#### 3.4.1 English long-lag plosives

The effects of vowel duration and place of articulation on MidDiff and HiDiff in English are given in Table 6.

**Table 6.** Linear Mixed-Effects Models of Relative Amplitude of English Long-Lag Plosive Bursts in (A) the F2/F3 Region (MidDiff) and (B) Above 3–3.5kHz (HiDiff), as a Function of Vowel Duration and Consonant Place of Articulation.

A: MidDiff					B: HiDiff			
Predictor	β	*SE*	*df*	*t*	β	*SE*	*df*	*t*
(Intercept)	40.03	1.48	2,033	27.02**	46.93	1.35	2,033	34.64**
POA-t	−11.18	0.60	2,033	−18.71**	−22.88	0.61	2,033	−37.77**
POA-k	−16.60	0.62	2,033	−26.59**	−12.43	0.63	2,033	−19.62**
Vowel duration	−45.40	2.97	2,033	−15.27**	−52.33	5.30	2,033	−9.87**
POA-t × Vowel dur	31.59	3.13	2,033	10.09**	51.46	3.18	2,033	16.21**
POA-k × Vowel dur	21.97	3.15	2,033	6.96**	33.82	3.21	2,033	10.55**

*Note*. Reference level is POA-p.

** *p* < 0.001.

**Figure 5.**
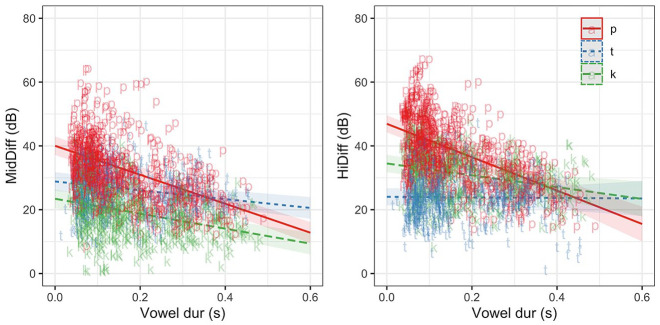
Predicted estimates (with confidence intervals) of MidDiff and HiDiff as a function of place of articulation and vowel duration in long-lag English plosives.

All fixed effects and interactions were significant as were the vowel duration interactions with alveolar and velar places of articulation (relative to bilabials). That is, the effect of vowel duration on the burst amplitudes of alveolars and velars is different from vowel duration effects on the burst amplitudes of bilabials. These interactions are evident in the model estimates shown in Figure 5.

The overall effect of place of articulation on burst amplitude measures are consistent with those given in Stevens et al. (1999) and correspond to peak *P*_o_ given in Subtelny et al. (1966), with bilabials having the highest difference measures (lowest amplitude relative to the vowel) in both regions of the spectrum (MidDiff = 40.03 dB; HiDiff = 46.93 dB).

Velars have a high-amplitude mid-frequency prominence (MidDiff = 23.43 dB) and a high-frequency prominence that is between bilabials and alveolars in amplitude (HiDiff = 34.5 dB). Alveolars have a mid-frequency amplitude higher (MidDiff = 28.85 dB) than bilabials, and a high-amplitude, high-frequency prominence (HiDiff = 24.05 dB).

#### 3.4.2 Tamil short-lag plosives

The effects of vowel duration and place of articulation on MidDiff and HiDiff in Tamil are given in Table 7.

**Table 7.** Linear Mixed-Effects Models of Relative Amplitude of Tamil Short-Lag Plosives in (A) the F2/F3 Region (MidDiff) and (B) Above 3–3.5kHz (HiDiff), as a Function of Vowel Duration and Consonant Place of Articulation.

A: MidDiff					B: HiDiff			
Predictor	β	*SE*	*df*	*t*	β	*SE*	*df*	*t*
(Intercept)	46.87	3.47	1,441	13.50**	52.50	3.73	1,441	14.06**
POA-t⊓	−5.73	1.33	1,441	−4.32**	−14.00	1.57	1,441	−8.94**
POA-ʈ	−16.13	1.41	1,441	−11.47**	−13.70	1.59	1,441	−8.62**
POA-k	−21.55	1.39	1,441	−15.55**	−13.17	1.50	1,441	−8.78**
Vowel duration	−23.01	9.73	1,441	−2.36*	−8.21	11.64	1,441	−0.70
POA-t⊓ × Vowel dur	−9.93	6.13	1,441	−1.62	−3.11	7.17	1,441	−0.43
POA-ʈ × Vowel dur	1.12	6.48	1,441	0.17	−16.57	7.32	1,441	−2.26*
POA-k × Vowel dur	5.60	6.35	1,441	0.88	2.61	6.93	1,441	0.38

*Note*. Reference level is POA-p.

** *p* < 0.001; *p < 0.05.

All fixed POA effects were significantly different from the reference bilabial. Similar to the long-lag English data, and consistent with Stevens et al. (1999), bilabials had the lowest burst amplitudes in both mid- and high-frequency ranges (MidDiff = 46.87 dB; HiDiff = 52.50 dB). Velars had the highest burst amplitude in the mid-frequency range, (MidDiff = 25.32 dB) and retroflexes followed by dentals had the highest burst amplitude in the high-frequency range (HiDiff_ret_ = 38.80 dB, HiDiff_den_ = 38.50 dB), again consistent with Stevens et al. (1999) which showed alveolars with the highest amplitude in that range.

**Figure 6.**
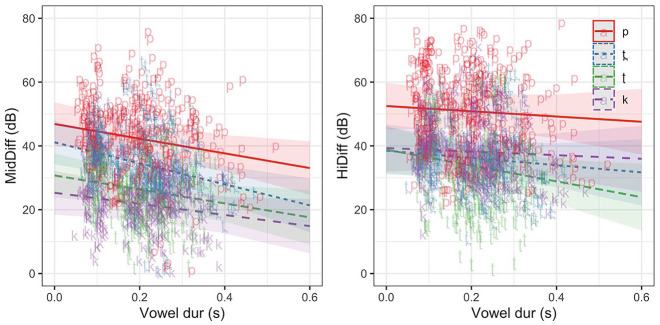
Predicted estimates (with confidence intervals) and observed values of MidDiff and HiDiff as a function of place of articulation and vowel duration in short-lag Tamil plosives.

Figure 6 shows the model estimates of the burst amplitudes for each place of articulation as a function of vowel duration. The vowel duration effects on the mid-frequency burst amplitudes were similar at all places of articulation (i.e., there are no significant interactions). While the overall effect of vowel duration on high-frequency burst amplitudes was not significant (*t* =−0.70, *p* = 0.48), retroflex stops showed a significantly steeper increase in amplitude with the increase in vowel duration relative to bilabials.

### 3.5 VOT, vowel duration, and burst amplitude in English and Tamil

**Figure 7.**
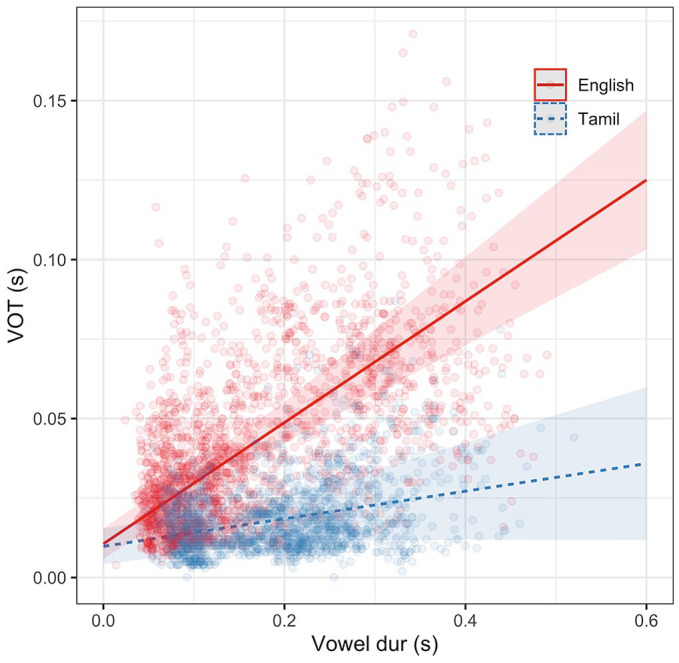
Predicted estimates (with confidence intervals)mixed-effects models and observed values of VOT in (A) long-lag English and (B) short-lag Tamil plosives.

To better understand the oro-laryngeal timing differences in burst amplitude as a function of speaking rate, the relationship between VOT and vowel duration was modeled in the two languages. The results of the VOT model are given in Table 8 and estimates visualized in Figure 7.

**Table 8.** Mixed-Effects Model of VOT as a Function of Vowel Duration in Long-Lag English and Short-Lag Tamil Plosives.

Predictor	β	*SE*	*df*	*t*
(Intercept)	0.01	0.002	3,484	4.35**
Vowel duration	0.19	0.02	3,484	9.19**
Language_Tamil_	−0.001	0.004	20	−0.22
Vowel dur Language_Tamil_	−0.15	0.03	3,484	−4.75**

*Note*. Reference level is Language_English_.

** *p* < 0.001.

Vowel duration has a clear positive effect on VOT. This result was consistent with literature showing that as speaking rate decreases, vowel duration and VOT increase in equal proportions (e.g., Kessinger & Blumstein, 1997; Theodore et al., 2009), as well as the *laryngeal realism* literature, suggesting that phonetic cues to phonological categories decrease in duration as speech rate increases (Beckman et al., 2011). The effect of vowel duration on VOT is different between the two languages, with short-lag Tamil plosives having a shallower slope.

Although there is an overall positive association between speaking rate and VOT, the size of the effect is reduced in Tamil short-lag implementation of VOT (75% of VOTs are less than 0.025 s), while the long-lag VOT status in English allows for a more flexible oro-laryngeal timing. Given the relative immutability of VOT in short-lag Tamil plosives, does it contribute to burst amplitude variability? The effect of VOT on burst amplitude measures were modeled separately in both languages.

### 3.6 VOT effects on burst amplitude

#### 3.6.1 Long-lag English plosives

Mixed-effects models of MidDiff and HiDiff were fit to the data with VOT as a predictor and random intercepts and slopes for VOT by subject. Model coefficients are shown in Table 9.

**Table 9.** Linear Mixed-Effects Models of Relative Amplitude of Long-Lag English Plosive Bursts in (A) the F2/F3 Region (MidDiff) and (B) Above 3–3.5 kHz (HiDiff), as a Function of Voice Onset Time.

A: MidDiff					B: HiDiff			
Predictor	β	*SE*	*df*	*t*	β	*SE*	*df*	*t*
(Intercept)	34.47	1.59	2,037	21.68**	38.77	2.08	2,037	18.57**
VOT	−192.92	21.12	2,037	−9.13**	−172.40	31.31	2,037	−5.51**

** *p* < 0.001.

The results show that VOT is correlated with burst amplitude in both the mid-frequency and high-frequency prominences. Similar to vowel duration, with the increase in VOT, burst amplitude increases (difference measures decrease).

#### 3.6.2 Short-lag Tamil plosives

Mixed models were likewise fit to the Tamil short-lag burst amplitude data, with VOT as a predictor and random slopes and intercepts for VOT by subject. Table 10 shows the model coefficients, which suggest a significant positive effect of VOT on burst amplitude in only the mid-frequency prominence, while the effect is muted (*t* =−1.83, *p* = 0.07) in the high-frequency prominence.

**Table 10.** Linear Mixed-Effects Models of Relative Amplitude of Tamil Short-Lag Plosives in (A) The F2/F3 Region (MidDiff) and (B) Above 3–3.5 kHz (HiDiff), as a Function of Voice Onset Time.

A: MidDiff					B: HiDiff			
Predictor	β	*SE*	*df*	*t*	β	*SE*	*df*	*t*
(Intercept)	36.78	3.81	1,447	9.64**	43.13	4.17	1,447	10.33**
VOT	−344.95	100.57	1,447	−3.43**	−185.01	101.13	1,447	−1.83

** *p* < 0.001.

The overall temporal constraining of the Tamil syllable (by increasing speaking rate, which minimally decreases VOT) primarily affects the mid-frequency burst amplitude. That is, the effects of VOT are comparable to the effects of vowel duration on Tamil short-lag burst amplitudes (Section 3.4).

## 4 General discussion

Linguistic factors such as syllable structure (Mackay, 1974) and phrasal length (Yuan et al., 2006), and extra-linguistic factors like emotional content of the speech (Mozziconacci & Hermes, 2000), and age and geographical background (Quené, 2005) of the speaker all have an effect on speaking rate. The current study investigated whether speaking rate variation imposes constraints on the articulation (and consequently aerodynamics and acoustics) of consonants via changes in burst amplitude, a proxy for intraoral pressure and glottal frication. Speaking rate (as evidenced in vowel duration) affected burst amplitude, such that decreasing vowel duration led to a decrease in amplitude (relative to the following vowel) in speakers’ consonant bursts. Although the effect is found in both long-lag (North American English) and short-lag (Indian Tamil) plosives, it is more pronounced in the long-lag series, as revealed by its interaction with place of articulation.

The results suggest that burst amplitudes of bilabials were lower in short-lag (Tamil) than in long-lag (English) plosives; the effect was smaller for velars. These differences were unpacked by examining the association between vowel duration and VOT. Consistent with the extant literature, long-lag VOT is more tightly associated with vowel duration when intended speaking rate increases or decreases. Although there is a relationship between vowel duration and VOT in short-lag plosives (as a function of rate adjustments), it is considerably more shallow. This suggests that the differing oro-laryngeal timing in the two types of plosives may be responsible for the burst amplitude variation. Long-lag VOT is strongly correlated with vowel duration as well as higher overall burst amplitudes than in short-lag plosives. When coupled with the general tendency for voiceless aspirated stops to have long closure durations (Lisker, 1957; Stathopoulos & Weismer, 1983), it can be deduced that the high burst amplitude results from sufficient time for *P*_o_ buildup. Conversely, short-lag stops have lower burst amplitudes than English (suggesting lower *P*_o_), with less pronounced effects of speaking rate. However, the automatic relationship between short-lag VOT and burst-amplitude need not necessarily follow in a way exemplified in the present data. For example, Korean fortis stops show high *P*_o_ and have burst energy comparable to lenis stops despite having short-lag VOTs (Cho et al., 2002). This suggests that the correlation between short-lag VOT, low *P*_o_ and low burst amplitude may be an articulatory-aerodynamic default when there is no phonological laryngeal contrast for stops in initial position.

### 4.1 Conclusion and future directions

We can conclude from this study that speaking rate, as manifested in vowel duration, affects the burst amplitude of preceding consonants, and that VOT interacts with that effect. These results are very much in line with a growing phonetics literature focused on rate variability and its contribution to phonological lenition (Kirchner, 2004; Priva & Gleason, 2020; Warner & Tucker, 2011), which has identified fast speech as affecting shorter duration and increased intensity of the periodic portion of intervocalic consonants. Lenition of consonants in these environments, which are often produced without a release burst or complete closure, is characterized by more intense sonorous low-frequency energy. The comparable measure in the current study of prevocalic consonants is decreased energy concentrated in the high-frequency bands of the spectrum. Both of these results are consistent with increased speaking rate resulting in essentially weaker consonants.^1^

While the outcome of fast speech in the intervocalic consonant literature is a non-phonologized synchronic lenition in production, directions for future research building upon the present results might consider how the acoustic variability (resulting from either speaking rate or oro-laryngeal timing) in consonant bursts contribute to both our understanding of (1) general consonant perception and (2) listener-driven diachronic patterns in languages.

From a purely speech processing perspective, we might ask about the extent to which the phonetic association between vowel duration and burst amplitude is represented in the listener, that is, does a listener expect an association between vowel duration (rate-conditioned or otherwise) and reduced burst amplitude? If so, how does a listener’s language experience affect their perception of this trading relationship (Repp, 1982)? Would the association between rate/vowel duration and burst amplitude be stronger long-lag than in short-lag plosives? Does the relationship serve as a perceptual cue for phonological voicing in English speakers (or other languages that contrast long- and short-lag VOT)? This line of research will contribute to not only our understanding of cue integration in consonant perception but also theories that center the role of experience with speech in individuals’ perceptual capacities.

A second line of inquiry considers the laryngeal-timing and place effects observed in the present study as contributing to our understanding of diachronic lenition processes. Although burst characteristics are part of a larger constellation of acoustic cues utilized by the listener in determining consonant identity, disruption of burst cues results in a greater reliance on formant transitional cues in perception (Dorman et al., 1977). When coupled with the tendency for speaking rate–induced adjustments to formant transition patterns (Duez, 1992; Gay, 1978; Krull, 1989) we offer the possibility that low burst amplitude resulting from either language-internal forces or external factors like speaking rate, may potentially be misperceived by the listener (Ohala et al., 1981). Are listeners more or less sensitive to amplitude changes in different frequency bands (i.e., is a reduction in mid-frequency noise as observed in the current short-lag data significant for the listener) at different places of articulation? Do patterns in reduced burst amplitude perception, especially in naturally low-amplitude voiceless short-lag bilabials, resemble the types of historical lenition changes found in languages (e.g., debuccalization of bilabials in languages like Old Kannada and Japanese; Gai, 1946; Sasaki, 2008)?

In this way, the study of naturally conditioned variation in stop consonant burst amplitude may allow us to better understand the nature of listener knowledge as well as how that knowledge potentially affects historical phenomena.

## Notes

1. Thanks to the reviewers for noting this important point.
